# Where Does Open Science Lead Us During a Pandemic? A Public Good Argument to Prioritize Rights in the Open Commons

**DOI:** 10.1017/S0963180120000456

**Published:** 2020-06-05

**Authors:** BENJAMIN CAPPS

**Keywords:** COVID-19, open science, open commons, community of rights, capitalist platforms, neoliberalism, public good

## Abstract

During the 2020 COVID-19 pandemic, open science has become central to experimental, public health, and clinical responses across the globe. Open science (OS) is described as an open commons, in which a right to science renders all possible scientific data for everyone to access and use. In this common space, capitalist platforms now provide many essential services and are taking the lead in public health activities. These neoliberal businesses, however, have a problematic role in the capture of public goods. This paper argues that the open commons is a community of rights, consisting of people and institutions whose interests mutually support the public good. If OS is a cornerstone of public health, then reaffirming the public good is its overriding purpose, and unethical platforms ought to be excluded from the commons and its benefits.

## Introduction

For some, the ongoing Severe Acute Respiratory Syndrome Coronavirus 2 (SARS-Cov-2) pandemic has “reaffirmed the urgent need for a transition to open science.”^1^ “The real antidote to epidemic is not segregation, but rather cooperation,”^2^ so that open science will accelerate societal and economic progress.^3^ Across the globe, open science (OS) is enhancing evidence-based nonpharmaceutical measures, and equitably contributing to scientific and clinical responses to the pandemic.^4^ Although OS has come to mean many things,^5^ this paper is a critique of the concept that all scientific knowledge resides in an “open commons.”^6^ OS is preferable to a competitive, secretive, and proprietary scientific culture;^7^ however, the veneration of data idealism under the “new” commons obscures potential abuse of OS, too.^8^

First, the pandemic has become a false panacea for OS in the unprecedented application of big data and fast science, leading to hurried and expedient publication, rather than prudent protocols, to support public health. Critics, however, point out that much of the information is not vetted, noisy, and can be socially and politically distorting. OS’s chaotic application is contributing to negative social determinants of fairness and equitability, in respect to whom the data is about, who can use it, and ultimately who controls it.^9^

Second, OS has become an end-in-itself, so its purpose to support *the public good* has morphed into *surveillance* that bleeds into social control and *profiteering.* Of these three OS models—respectively “public good,” “surveillance,” and “marketism”—only the first is part of an ethical open commons: surveillance intrinsically substantiates a conclusion that there are few communal values, and markets accentuate competition rather than cooperation. “Surveillance capitalism,” regardless, has blended into the commons architecture in the form of “capitalist platforms” such as Amazon, Apple, Google, and Facebook, as well as data analysis start-ups such as Palantir and Clearview AI. These businesses enable "value-creating interactions between external producers and consumers, providing an open, participative infrastructure for these interactions and setting governance conditions for them.”^10^ However, the interactions are organized around maximizing market goals and not public health ones, and their governance proves to be self-serving. Through their hold on big data, it is becoming much harder to avoid their power, yet they are often unregulated, lack transparency, and embody privacy norms different than those established in medicine or research. Despite these concerns, they have begun to provide essential services or public goods based on the epidemiological legitimacy of surveilling peoples’ health, and are being positioned to lead some aspects of government pandemic plans.^11^ OS, therefore, has also become a contingency for irresponsible innovation, research misconduct, and political interference across the open commons “… that threatens the existential and political canon of the modern liberal order defined by principles of self-determination that have been centuries, even millennia, in the making.”^12^

At a time of loss, hardship, chaos, and uncertainty, where does the OS narrative lead us? In the following, I caution that the COVID-19 pandemic has become an opportunity for surveillance capitalism, and its entrenchment through some initiatives could permanently change the open commons to a “new normal” that jeopardizes our rights. OS imagines an open commons of equally beneficent participants, donors, altruists, and users; but the *market* value of OS has become the “distributed and largely uncontested new expression of power” or the “Big Other,” as Shoshana Zuboff calls it.^13^ That value has become a characteristic of behavioral prediction and its modification under a pretext of public health. An ethical commons, therefore, must be wary of platforms pretending to avert, or actively exploiting, gaps in fundamental ethical, legal, and social foundations of OS.

After first outlining the concept of OS, I consider three phases that led to the emergence of the current open commons. First, a specific innovation-based critique of autonomy underlies an uncritical celebration of OS potential for vast social and economic value.^14^ That view is mostly naïve to the emergence of the neoliberal capitalist platforms. The second phase explains how the idea of the open commons developed, but that view continues to be incredulous of the impacts of neoliberal ideology, for instance, by allowing conflation of research *integrity*—a condition which is essential during this pandemic emergency—^15^ with the *innovative* purposes of an underregulated market. The third phase has strengthened conditions for an ethical commons under a “right to science,” but its belief in untrammeled obligations to science does not exclude divisive industries or purposes that risk undermining public health.^16^

I argue that if the open commons is a community of rights, consisting of people and institutions who are mutually “for the public good,” then it is a mistake to hold OS to be entirely inclusive.^17^ In the final section, I argue that the open commons demands stronger normative principles to support innovative use of new scientific knowledge (in this paper, I use data and information interchangeably), but also requires obligations to use it ethically. Focusing on the question of markets (rather than surveillance), this paper attempts to keep OS as a cornerstone of public health, and therefore reaffirms the public good as OS’s overriding purpose.

## The Concept of Open Science

During this pandemic, OS has become central to sharing data about the SARS-CoV-2 and clinical nature of COVID-19. This exponential growth of real-time information has been used directly to justify public health policies. OS is not new to this pandemic, however, and its narrative has a distinct ideology which could have an impact on how we emerge from this time.

The contemporary idea of OS comes from a nebulous background: it has origins in history,^18^ social convention,^19^ jurisprudence,^20^ and ethics.^21^ These ideas represent different heritages about what constitutes favorable conditions for social innovation. Today, OS is principally positioned as a response to the unprecedented production of data—too quickly to be contained and too much to be constrained by solitary users—and a realization that misuse of proprietary models can discourage socially valuable innovation.^22^ OS, therefore, began the gradual normalization of open access journals as a benchmark for scientific dissemination, and has since become a movement to advocate for a new “open commons” to underpin all parts of the research process. Colloquially, OS is about removing barriers to scientific knowledge, and, as such, it has supported creative models that prioritize better and faster access to science for anyone who wants to use it. OS is meant to remove structural obstacles to reporting and dissemination, and thereby optimize socially valuable practices (e.g., “open access,” “open data,” and “open source”).^23^ Numerous funding agencies and governmental institutions stipulate ethical conditions like transparency and fairness, confident that opportunities stemming from OS will be socially equitable.^24^ Some industry-based individuals and institutions have integrated OS into their work ideology.^25^ It is also anticipated that publics, such as users, research participants and patients, will share their data too.^26^ To this end, OS is interconnecting all domains of scientific record-keeping, archiving, discovery, and innovation, and has taken root in the community ethos underlying citizen science and crowdsourcing.

Primarily, OS maximizes efficiencies in the knowledge economy by reducing proprietary claims on intellectual property and promoting co-created knowledge. Doing so enhances international and interjurisdictional cooperation on fair data creating, access and use. There is evidence that OS benefits researchers through recognition, partnerships, and enhanced data access. It ensures critical review and reproducibility, and opens up scientific culture to social scrutiny and thereby reinforces the scientific method (i.e., advocating holistic creation and stewardship of knowledge).^27^ OS may become a cornerstone in clinical practice, enabling diagnostics and cures using patient records across vast places and time.^28^ In public health, real-time data analytics enables rapid and equitable responses to pandemics.^29^

Although OS culture may be a result of compromises, paradoxes, and surprises, it now seems to be the latest bandwagon; yet the reasons to jump on it may not always be compelling. In the context of this paper, OS raises concerns about the contexts it creates for excessive scope for monitoring^30^ and control (e.g., classification, profiling, and ultimately manipulation).^31^ OS—premised on big data—also opens up many other controversies^32^: the data is messy, noisy, and often irrelevant, and creates ideal conditions for creating falsehoods and misleading perceptions to influence public policy. That raises further challenges for legitimate evidence, especially in situations where rapid publication compounds long-standing problems such as effective peer review and scrupulous communication of science.^33^ By necessity, technology-based sociality (the online world) forces us to leave our digital footprints everywhere; these can be (falsely) incriminating or exonerating, embarrassing, and harmful-if-known. It is difficult to hide or cover those tracks, and the millions of data points they reveal are easily harvested^34^ and assimilated into research without consent.^35^ Patients lose control of data, as it is sequestered under conditions that refuse further access, and patients are denied use of, or exploited for access to, end products.^36^ *Bona fides* scientists may be held responsible for disingenuous use of their data, and experience the backlash when someone else openly gets it wrong.^37^ The conditions for OS can theoretically preclude ethical scientists opportunities if they are concerned about specific circumstances of how data will be used; moreover, the imperative to take part denies researchers control over their hard worked-for data, and also makes them obedient to signals from the market. This milieu simultaneously undermines and celebrates “experts” and displaces scientific professionalism and the scientific method: experience and qualifications can be replaced by familiarity with particular forms of social criticism and popular debate, and therefore scientific authority and professional responsibility are won or lost depending on ideology. Strategic promotion of the “citizen scientist” also makes a mockery of their years of training, and questions experts’ claims to have authority.^38^

Finally, in the open commons, as we shall see, data created under ethical conditions for the public good, such as medical health records, can be acquired under a social pretext, capturing its benefits for traditional and novel market economies.^39^ These possibilities erode the ethos of communal science.^40^ The capitalist platforms seemingly leading this are knowingly impacting on public health: their acquisition of data relies on freeriding the open commons, using OS to create a competitive advantage, but then implementing proprietary rules to protect their interests.

## Open Science Ethics

In the earliest of at least three phases, OS emerged as a movement to maximize practical conditions for fruitful collaborations and enhanced sharing. Proponents talked about unprecedented knowledge generation and imagined that incredible transformative discoveries were imminent.^41^ This new way of doing science would be transformative, so it became necessary to define ethical conditions for data deposit, access, and use. One of the key challenges became the tension between maximizing innovation, on the one hand, and respecting autonomy, on the other. The argument turned out to be comparable to the utilitarian reasoning underlying primitive ideas about public health, where collective wellbeing (a good in itself) may conditionally trump rights.^42^ Thus, OS proponents appealed to “the public interest” to explain an innovation-based critique of autonomy that justified rescinding rights for the greater good.^43^ In this respect, proponents may acknowledge the problems of the unproven OS research paradigms practiced by capitalist platforms, but nevertheless remain positive about the capacity for society and jurisprudence to evolve an appropriate balance, that is, in respect to promoting innovation, but still having expectations about privacy and confidentiality. They argue that research participants and patients must adapt to this sea change too, despite the risks to their rights, because on balance they benefit from the innovative opportunities of OS.^44^ This view, however, shares the commercial function of “capitalist platforms,” and therefore cannot stop economies from undermining the communities they claim to serve.

“Social openness,” illustrated by uninhibited social media use, has increasingly normalized capitalist platforms.^45^ All the while, these platforms have gained further footholds in providing essential social services, often changing them into devices of capitalism. Within OS, there have been few questions raised as to whether these business models provide the most efficacious approach to data management in public health circumstances,^46^ and some have forsaken concerns about the ethical appropriateness of private interests taking part in providing public goods.^47^ OS, in fact, creates a self-sustaining context for limitless data, and that data has become extraordinarily valuable now that the mechanisms for extraction and exploitation are in place: big data is a naturally, freely, and effortlessly self-propagating good, requiring little more than strategic mining.^48^ The “new oil” has become ascendant as a unit of capital for market engines.^49^ In this respect, the “extreme concentration of wealth means an extreme concentration of power,” so that the capitalist platforms have become extraordinarily influential.^50^ As our wellbeing has increasingly shifted online, that space has become a progressively attractive one for enhancing entrepreneurial freedoms, extending beyond core businesses (social networking and e-commerce) into providing basic services such as health care. Meanwhile, there has been a global capitulation to neoliberal values, presenting an opportunity to keep the state in a subservient role of merely preserving institutional frameworks appropriate to market practices.^51^ Free market capitalists therefore imagine OS supports *laissez-fairism*: it lacks government interventionism (i.e., strict control over data) so is conducive to the kinds of liberalization that promotes capital generation.^52^ OS also organizes society through voluntary and community activity, cooperation, civicness, networking, and social capital: these are easily exploited for their production of vast and free data. So, if there is any truth to a sociological view that “privacy is dead,”^53^ then OS is an ideally “lawless” space to capture the public good.^54^ The social work is freely done, allowing the platforms siphon off a rent from every transaction they facilitate.

This neoliberal ruse is exemplified in a critique of “Surveillance Capitalism”^55^ or “Dataveillance”^56^: the “unexpected and illegible mechanisms of extraction and control that exile persons from their own behavior.”^57^ Although many remain wary of the surveillance narrative (what will health apps be used for after the pandemic?), we seem less concerned with those who opportunistically clearcut the public good through its capture, using an “inherent political asymmetry …[and] in fact *a posteriori* private expropriation.”^58^ OS risks opening the door to exploitative practices,^59^ conflicts of interest,^60^ and poor data security,^61^ under a vague conjecture about the public interest.^62^ The potential consequence of doing so takes us further down the path to dystopia: we become imprisoned but “happy consumers”—*homo datus* or data avatars, content (perhaps) to be counted, analyzed, and surveyed.^63^ Big data, bioinformatics, and AI combine to create artificial identities, replacing our dignity with a price to know everything about us. In this form, persons have insufficient knowledge about what is known about them and little ability to control how it is used.^64^ The problem is not necessarily the monolith of state or the forces of innovation, but them acting together in a neoliberal adaptation of the role of public health. In so doing, public health now serves a public interest in strong economies. Seen this way, OS makes communities more surveilled and potentially less free, paradoxically at odds with the intent of the open commons.^65^

## Exploiting the Open Commons

Perhaps as a result of the blurring of public health and capitalist agendas, in the second phase of OS, proponents have organized the movement into an “open commons.”^66^ The open commons has become more than the aggregation of data; it shifts OS arguments from “intellectual property or confidentiality restrictions,” to the “fundamental shared nature of the genomic commons.”^67^ Given the right context, there is an ethical obligation to take part in these communities situated in both research and healthcare contexts. However, we are permanently connected through our household economies, education, work, use of the health system and social services, and all our real and virtual socializing. These circumstances perhaps entertain a contemporary “right to the internet,” especially when the circumstance of a pandemic befalls a society. The open commons therefore signifies a continual connection between our being and with *very large data,* both created spontaneously (i.e., by social media use, as well as other nonprofessional activities) and by structured initiatives such as health care, biobanks and specific analytic websites, for example, genetic and virological. The data is shared between traditional networks of local, regional, and international research infrastructure and hubs,^68^ as well as real-time database apps, platforms, and archives.^69^ Thus, following Elinor Ostrom, the open commons has become a massive “common pool resource” rather than a public good; data is a resource that *may be decreased* through consumption, and *exclusion is possible*, necessitating complex (and ethical) rules for deposit, access and use.^70^

This “New Commons” still presupposes a community coopted for the good of science, but its people are compelled to give up some of their interests without expecting immediate gains or fearing instantaneous harms.^71^ This may be explained by an antecedent view—which contains elements of Robert Merton’s “Communism”—that a culture of sharing underlies all fruitful collaboration and equitable transfer of legitimate goods between citizens and scientists. For example, the total aggregation of accessible genomic data (across many institutions and resources) is referred to as the “genomic commons”^72^: that is a community of liberal citizens and public scientists working with their industrial counterparts, who are equally committed to the values of OS.^73^ In this respect, OS fosters behavioral change in those who habitually conceal their data. In reality, these relationships require “tiered access” that assesses individuals or organizations, and grants them specific data-use conditions.^74^

The commons, however, has become a place to influence social and political discourse. For example, the case may be made that if the open commons is in the “best interests” of people,^75^ then it also creates a space for the specific interests of a sector that stands to profit from exploiting its co-inhabitants.^76^ However, neoliberalism also applies to the kinds of entrepreneurial “experiments” currently undertaken in the open commons. A particular example is the emergence of “open research”—often bypassing ethics scrutiny^77^—that blurs principles of research integrity with the social and economic critiques of big data. Big data research not only uses data voluntarily provided (sometimes) and spontaneously harvested (with or without persons’ consent), but its researchers have no qualms about using data that is secretively or disingenuously gathered, because there is an competitive advantage in doing so and it comes with few penalties (and powerful advocates).^78^ In the background to these researches, there is an unfettered market where neoliberals may opportunistically capture public resources: commodification transforms a market economy into a market society, in which the solution to all manner of social and civic challenges is “the market” itself.^79^ That is more worrisome, because the OS consensus generally falls on publics to be players, and public institutions to support it, rather than being obligatory or reciprocal on the private sector.^80^ In that regard, production of data has complex, mostly public but sometimes private origins, so that OS idealism may be used to deliberately weaken those institutions operating “for the public good.” Doing so creates ideal circumstances to generate *public bads* that prospectively obstruct or reduce social opportunities: captured goods may become disruptive commodities.^81^ In the current narrative of OS, privacy may not be the only right we stand to lose by the subversion of the commons,^82^ as access and use of both legitimately and surreptitiously obtained data^83^ not only affect persons’ freedom and wellbeing, but may undermine the commons by promoting illegitimate interest. These possibilities stoke criticisms about the corporate abuses of data,^84^ which could weaken our response to the Covid-19 pandemic and ultimately reduce the effectiveness of public health.

## Taking a Different Path: A Rights-Centered Open Commons?

In this third phase, proponents have attempted to move away from utility-based arguments, to define a more complex ethical environment that frames OS as a “human right to benefit from the fruits of scientific research.”^85^ Significantly, our existence in the commons is both essential (as sociable, cooperative beings) and unavoidable (as perpetually online beings), so that its moral governance requires a rights framework. In this respect, the “right to science” includes an untrammeled obligation to share, and a social contract involving trade-offs that are neither necessarily mutual or correlative, so that the open commons becomes critical, as well as supportive, of the conditions for freedom and wellbeing.^86^ Finding out where that right sits between autonomy and the public interests requires tracing it back to its roots in international conventions, where we find that right is anchored to equal dignity, self-realization, and substantive freedoms.

Thus, the right to science *must* include the right to consent to scientific experimentation. That right is fundamental to the integrity of science, and the development and diffusion of ethical technologies; that is, it protects persons from science misuse.^87^ The “right to science” is simultaneously a veneration of good knowledge as a “public good”—a resource for the realization of human rights^88^—and also limited by fundamental obligations to “human dignity.”^89^ The correlation of the public good and dignity inevitably creates a tension between “the public interest” and autonomy; but since recognizing the atrocities of mid-20th century committed by doctors and scientists, autonomy has been clearly favored over the public interest. Without that correlate, the right to science, as a disambiguation of an obligation to share, yet lacking stable protections of freedom and wellbeing, is on shaky ground.^90^ The ground becomes firm only if the right to science is within a hierarchy of obligations. Foremost there is the protection of basic rights (as formulated, e.g., in the Nuremberg Code), which later evolved to include a right to receive reasonable technological benefits. The basic rights create a correlative obligation to a *prima facie* positive right to privacy as well as a negative right to be “left alone.”^91^ Next, the public interest promotes rights in the sense of general welfare—that is what tells us what is good about the commons—and promotes opportunities to enjoy second tier rights, proportional to the tension between diminished freedom and prospectively enhanced wellbeing.^92^ Last, there is a right to engage in ethical science. Although we may all “enjoy the benefits” of science, it is clear that citizens, whether they take part in its creation or revel in its progress, also have a choice in both respects; that choice is a freedom from an unjustified public interest.^93^

What I have just described in brief terms is a “community of rights,” in which an egalitarian conception of solidarity promotes OS as mutual and cooperative, rather than secretive, manipulative or competitive.^94^ Therefore, the kinds of research conducted in this space must be *bona fides*, which excludes activities that prospectively obstruct or reduce social opportunities (i.e., public bads) and precludes capture of public resources. The ethical commons, therefore, is conditional on the technical examination of “the public interest” in terms of the public good and legitimate rights.^95^ There can be no duty to take part in OS without careful consideration of the public good; and, as such, we can reasonably opt out of taking part in research that foreseeably harms us.^96^ Moreover, the public interest creates obligations for institutions: innovation is an ability to make use of new scientific knowledge, but also a capacity to put it to use creatively and ethically to help solve broadly social problems. In general, candidate institutions must first commit to a normative *principle of institutional responsibility.* ^97^ This principle stipulates that they observe the meaning of the public interest as an “indirect” form of a social contract to promote the public interest in welfare.^98^ Such an application of rights theory means that they are practically as committed to equally respecting equally the rights of people in the commons as they are to their shareholders. Responsible institutions protect rights and interests jointly by including procedural requirements for ethical associations and partnerships and establishing instrumental governance.^99^ All this requires a regulatory response focused on the OS sector as a whole, since it is not always easy to separate research or practice into distinct private, public or not-for profit forms.

Institutional responsibility also requires that public–private industry occupancy of the commons be ethically symmetrical. The onus also falls on prospective partners to provide reciprocal openness, so that its intentions become transparent in respect to why it collects data and what it is used for.^100^ Ethically, participating private industries must contribute corporate data to the public good, too, just as legitimate public bodies do. Private institutions should compensate the open commons, so as to preclude freeriding on costs incurred by other people; that premium can be adjusted in respect to their adherence to these conditions. Finally, these conditions should be spelled out in specific “data collection” and “data use” rules that are externally enforced, and there should be oversight in respect to applying norms of research ethics.^101^

We can learn a lot from nuanced approaches to OS such as that of UK Biobank: it stipulates that its purpose is *for the public good,* so that its stewardship over data is conditional. The data it contains is never truly open, but is accessible to all *bona fides* researchers, whether public or private, on the conditions that data is returned to the resource and “unreasonable” patents are precluded in any future invention.^102^ Like UK Biobank, the open commons may exclude those who attempt to capture goods or create public bads.^103^ Alas, although there are industries volunteering to use this approach already, in general joining the commons for them likely requires a comprehensive sea change to alter course from a speculated social cataclysm of post-pandemic capitalism.^104^

## Conclusion

The current OS narrative potentially underestimates the opportunities for surveillance capitalism during a pandemic. Enthusiasm for innovation, the open commons, and the “right to science” continue to conflate ethical conditions for *bona fides* research with capital purposes. This mistake has become an opportunity to undo rights protections, as OS proponents cannot set effective ethical conditions for data access and use: OS avails all possible scientific data for *anyone and everyone* to access and use *for any purpose.* Ethical OS requires a far more cautious approach in respect to how society emerges from the pandemic: new data, as well as new technologies (i.e., AI) and tools (open source software), even if used ethically now, will become resources able to be used beyond the purposes, and protections offered by, public health.^105^ Despite trust in some legal safeguards,^106^ OS has also become an opportunity for capitalist platforms to provide many essential services based on public health’s legitimacy of surveilling peoples’ health. This paper, therefore, provides some evidence that the protections afforded to persons may be rolled back under the “new commons,” and that could undermine the essential provision of public goods. If we begin to imagine OS as simply knowledge generation *qua* innovation, without establishing ethical norms, as well as legal protections, the conditions for the public interest can quickly become ambiguous in respect to the public good. This conclusion may be resisted still, because there is likely little social appetite for returning to times when science was more of a proprietary activity. But it will not do to remain ambiguous or ambivalent to the influence of surveillance capitalism.

The open commons is not only data. It is a patient’s expectant diagnosis, a community that provides care and future welfare, and it is where worthwhile research is done to promote public health. It is also the social space where much of our lives has shifted to during the COVID-19 pandemic. These activities are underpinned by the public good, which establishes fundamental obligations on individuals and institutions that, at times like these, are necessary for an effective pandemic response.^107^ In the community of rights, the freedom of the commons is given to friendly participants, donors or altruists, and their exclusion from its benefits is unethical; people are free to come and go. The egalitarian response to a potential the tragedy of the commons, brought about by misplaced trust in capitalist platforms, is to exclude those that “follow strategies that destroy the very resource” itself;^108^ and recognition that the threat from open data use is “so sweeping that its can no longer be circumscribed by the concept of privacy and its contests.”^109^

The worthy ambitions of ethical OS need safeguarding by expanding the narrative to the existential challenges of our time; it has become evident that the emergent OS ecosystem is not sufficiently equitable, and encourages activities that actively reduce socially valuable outcomes. Past examples of the untrammeled use of data (most recently, in political campaigns) raise concerns about the extent of data held about persons, and how that data can be manipulated and in what ways and for what purpose. The current OS narrative does not go to the root of these concerns about the breadth of information needed and available to make sophisticated predictions about people, and ultimately, the consequential decisions made that limit their freedom during a public health crisis and beyond. A new sense of solidarity in the open commons is one of the few reassuring things to have happened during this pandemic, and through experiencing degrees of alienation, illness, poverty and sadness during the pandemic, communities should not be exploited by entities compelled by old-fashioned, anti-community ideas of neoliberalism.

## Notes

1. UNESCO. *COVID-19—Open Science and Reinforced Scientific Cooperation*; 2020; available at https://en.unesco.org/covid19/sciencesresponse (last accessed 8 May 2020).

2. Harari Y. In the battle against coronavirus, humanity lacks leadership. *Time*; 2020 March 15; available at https://time.com (last accessed 8 May 2020).

3.

Bountra C , Hwa Lee W , Lezaun J. A New Pharmaceutical Commons: Transforming Drug Discovery. Oxford: University of Oxford; 2017 .

4. Cyranoski D. Profile of a killer: The complex biology powering the coronavirus pandemic. *Nature* 2020;581:22–6.

5.

Caulfield T , Harmon S , Joly Y. Open science versus commercialization: A modern research conflict. Genome Medicine 2012;4:17 2236979010.1186/gm316PMC3392762.

6.

Contreras J , Knoppers B. The genomic commons. Annual Review of Genomic and Human Genetics 2018;19 10.1146/annurev-genom-083117-02155229400986:1.1–1.25.

7.

Capps B , Chadwick R , Joly Y , Mulvihill JJ , Lysaght T , Zwart H. Falling giants and the rise of gene editing: Ethics, private interests and the public good. Human Genomics 2017;11:20 2885144410.1186/s40246-017-0116-4PMC5575847.

8.

Powles J , Hodson H. Response to DeepMind. Health and Technology 2018;8:15–29 10.1007/s12553-017-0179-1PMC574178329308344. Griffin A. Coronavirus contact-tracing app could make ‘privacy and rights another casualty of the virus’, campaigners warn. *The Independent* 2020 May 5; available at https://www.independent.co.uk (last accessed 8 May 2020).

9. O’Neill P, Ryan-Mosley T, Johnson B. Covid tracing tracker. *MIT Technology Review* 2020 May 7; available at https://www.technologyreview.com/2020/05/07/1000961/launching-mittr-covid-tracing-tracker/ (last accessed 12 May 2020).

10. Weatherby L. Delete your account: On the theory of platform capitalism. *Los Angeles Review of Books* 2018 Apr 24; available at https://lareviewofbooks.org/ (last accessed 8 May 2020).

11. See note 9, O’Neill et al. 2020. These platforms see lax regulatory infrastructure, including rescinding privacy rights for mass surveillance (and unbounded data gathering), conducive to maximising further control by those best (and privately) positioned to do so; Klein N. Screen new deal. *The Intercept* 2020 May 8; available at https://theintercept.com/2020/05/08/andrew-cuomo-eric-schmidt-coronavirus-tech-shock-doctrine/ (last accessed 8 May 2020).

12. Zuboff S. The secrets of surveillance capitalism. *Frankfurter Allgemeine* 2016 Mar 5; available at https://www.faz.net/aktuell/ (last accessed 8 May 2020).

13. See note 12, Zuboff 2020.

14.

Goroff D , Polonetsky J , Tene O. Privacy protective research: Facilitating ethically responsible access to administrative data. Annals of the American Academy of Political and Social Sciences 2008;675:46–66 .

15. Marcus A, Oransky I. The science of this pandemic is moving at dangerous speeds. *Wired* 2020 Mar 28; available at https://www.wired.com (last accessed 8 May 2020).

16. The “fact that open science is proposed equally as a panacea for each and every one of these problems is what prompts the suspicion of snake oil”; Mirowski P. The future(s) of open science. *Social Studies of Science* 2018;48:171–203, at 173.

17. Gewirth A. *The Community of Rights.* Chicago: Chicago University Press; 1996, at Sec. 3.4. The “community of rights” presupposes conditions where the distribution of goods, based on their needfulness, impacts for better or worse on all those living in proximity to one another. In this regard, Roger Brownsword writes: “We can argue about the details of this context (or commons) but it will include elements pertaining to our wellbeing (clean air and water, food, environmental integrity, and the like) and our freedom (security, an absence of fear and intimidation, and so on).” Brownsword R. Friends, Romans, and countrymen: Is there a universal right to identity? *Law, Innovation and Technology* 2009;1:223–49, at 225.

18.

David P. The historical origins of open science: An essay on patronage, reputation and common agency contracting in the scientific revolution. Capitalism and Society 2008;3:5 .

19.

Merton R. The Sociology of Science: Theoretical and Empirical Investigations. Chicago: University of Chicago Press; 1973 .

20.

Kapczynski A. Order without intellectual property law: Open science in influenza. Cornell Law Review 2017;102 29048862:1539–648.

21.

Levin N , Leonelli S. How does one ‘open’ science? Questions of value in biological research. Science, Technology, & Human Values 2017;42:280–305 10.1177/0162243916672071PMC530208528232768.

22.

Vicente-Saez R , Martinez-Fuentes C. Open science now: A systematic literature review for an integrated definition. Journal of Business Research 2018;88 :428–36.

23.

McKiernan E , Bourne PE , Brown CT , Buck S , Kenall A , Lin J , et al How open science helps researchers succeed. eLife 2016;5:e16800 2738736210.7554/eLife.16800PMC4973366.

24. See note 6, Contreras, Knoppers 2018.

25. See note 6, Contreras, Knoppers 2018.

26. Frank O. Your health data helps everyone. *New York Times* 2019 Oct 3: 29, section A.

27.

Ariew R. Descartes and the tree of knowledge. Synthese 1992;92 :101–16.

28.

Wright C , Ware JS , Lucassen AM , Hall A , Middleton A , Rahman N , et al Genomic variant sharing: A position statement. Welcome Open Research 2019;4:22 10.12688/wellcomeopenres.15090.2PMC691321331886409.

29.

Coloma J , Harris E. Open-access science: A necessity for global public health. PLOS Pathogens 2005;1(2):e21. 1625459910.1371/journal.ppat.0010021PMC1266310

30. Cadwallader C, Campbell D. Revealed: Facebook’s global lobbying against data privacy laws*. The Observer* 2019 Mar 2; available at https://www.theguardian.com (last accessed 8 May 2020).

31.

Ohm P. Changing the rules: General principles for data use and analysis In Lane J , Stodden V , eds. Privacy, Big Data, and the Public Good: Frameworks for Engagement. Cambridge: Cambridge University Press; 2014, at 99–111 .

32. A similar process, it should be noted, has left persons without control over cells and tissues taken from their body; Capps B. Redefining property in human body parts: An ethical enquiry in the stem cell era In: Akabayashi A , ed. The Future of Bioethics: International Dialogues. Oxford: Oxford University Press; 2014 , at 235–63.

33. Glasziou, P. Waste in covid-19 research. *BMJ* 2020;369:m1847; Laakso M , Welling P , Bukvova H , Nyman L , Björk BC , Hedlund T. The development of open access journal publishing from 1993 to 2009. PLoS One 2011;6(6):e20961 2169513910.1371/journal.pone.0020961PMC3113847.

34.

Tsesis A. The right to erasure: Privacy, data brokers, and the indefinite retention of data. Scientific American 2014;49 :433–84.

35.

Capps B. Models of biobanks and implications for reproductive health innovation. Monash Bioethics Review 2015;33:238–572667068610.1007/s40592-015-0042-y.

36. For example, Ebola blood samples taken from patients during the 2014–2016 epidemic in West Africa: “while some ethicists argued that the public good that could emerge from a vaccine or cure outweighed researcher responsibilities to any of the patients, it reinforces a perception that patients—and particularly patients in the developing world—do not have any rights”; Freudenthal E. Ebola’s lost blood. *The Telegraph* 2019 Feb 6; available at https://www.telegraph.co.uk (last accessed 8 May 2020). Lerner S. Big Pharma prepares to profit from the Coronavirus. *The Intercept* 2020 Mar 13; available at https://theintercept.com/2020/03/13/big-pharma-drug-pricing-coronavirus-profits/ (last accessed 13 May 2020).

37.
*Bona fides* (Latin: honesty or lawfulness of purpose) indicates a systematic, investigative, or experimental activity conducted in good faith for the purpose of acquiring (and sharing) knowledge. It includes veracity in one’s good faith or genuineness, and evidence/testimony of one’s qualifications, reputation and achievements; Johnson H , South N , Walters R. The commodification and exploitation of fresh water: Property, human rights and green criminology. International Journal of Law, Crime and Justice 2016;44:146–162 ; Ritson S. Crackpots and active researchers: The controversy over links between arXiv and the Blogosphere. Social Studies in Science 2019;46 10.1177/030631271664750828948876:779–88.

38.

Capps B. The funding of medical research by industry: Can a good tree bring forth evil fruit? British Medical Bulletin 2019;118:5–15 10.1093/bmb/ldw014PMC512742227151955.

39.

Godlee F. What can we salvage from Care.data? BMJ 2016;354:i3907 .

40.

Capps B. Public goods in the ethical reconsideration of research innovation In: Capps P , Pattinson S , eds. Ethical Rationalism and the Law. Oxford, UK: Hart Publishing; 2016 , at 149–69.

41.

Knoppers B , Joly Y. Introduction: The why and whither of genomic data sharing. Human Genetics 2018;137 10.1007/s00439-018-1923-y30097718:569–74.

42. Compare with, for example, the “community of equals”; Dworkin R. Taking Rights Seriously. London, UK: Duckworth; 2004 , at 368.

43.

Deverka P , Majumder MA , Villanueva AG , Anderson M , Bakker AC , Bardill J , et al Creating a data resource: What will it take to build a medical information commons? Genomic Medicine 2017;9:1–5 10.1186/s13073-017-0476-3PMC561043228938910; Thorogood A. Canada: Will privacy rule continue to favour open science? Human Genetics 2018;137:595–602 3001418810.1007/s00439-018-1905-0PMC6132649. Also: “Yet in conventional public health practice, a paternalistic approach may prevail where attempts to change the behavior of the individual citizen are thought to be both acceptable and desirable’; Davies S. *Annual Report of the Chief Medical Officer 2016, Generation Genome.* London, UK: Department of Health; 2017 at 6.

44. Compare: Townsend D. Privacy, politeness and the boundary between theory and practice in ethical rationalism. In: Capps P, Pattinson S eds. *Ethical Rationalism and the Law.* Oxford, UK: Hart Publishing; 2017, at 171–90.

45. For example, in 2010, Mark Zuckerberg declared: “People have really gotten comfortable not only sharing more information and different kinds, but more openly and with more people… That social norm is just something that has evolved over time”; Johnson B. Privacy no longer a social norm, says Facebook founder. *The Guardian* 2010 Jan 11; available at https://www.theguardian.com (last accessed 8 May 2020).

46. Landau, S. 2020. Location surveillance to counter COVID-19: Efficacy is what matters. *Lawfare* 2020 Mar 25; available at https://www.lawfareblog.com/location-surveillance-counter-covid-19-efficacy-what-matters (last accessed 11 May 2020).

47.

Hallo De Wolf A , Toebes B. Assessing private sector involvement in health care and universal health coverage in light of the right to health. Health and Human Rights 2016;18:79–92 28559678PMC5394993; Morgan R , Ensor T , Waters H. Performance of private sector health care: Implications for universal health coverage. The Lancet 2016;388 10.1016/S0140-6736(16)00343-327358251:606–12.

48. Leaders. Regulating the internet giants: The world’s most valuable resource is no longer oil, but data. *The Economist* 2017 May 7; available at https://www.economist.com (last accessed 8 May 2020).

49.

Mayer-Schönberger V , Cukier K. Big Data: A Revolution That Will Transform How we Live, Work, and Think. Boston, MA: Houghton Mifflin Harcourt; 2013 .

50.

Saez E , Zucman G. The Triumph of Injustice: How the Rich Dodge Taxes and How to Make Them Pay. New York, NY: W. W. Norton; 2019 .

51.

Harvey D. A Brief History of Neoliberalism. Oxford: Oxford University Press; 2006 .

52. They “…skillfully exploited a lag in social evolution as the rapid development of their abilities to surveil for profit outrun public understanding and the eventual development of law and regulation that it produces”; Zuboff S. Big other: Surveillance capitalism and the prospects of an information civilization. *Journal of Information Technology* 2015;30:75–89, at 83.

53. Mance H. Is privacy dead? *Financial Times* 2019 Jul 19; available at https://www.ft.com (last accessed 8 May 2020).

54.

Emerson T. The System of Freedom of Expression. New York, NY: Random House; 1970 .

55. See note 12, Zuboff 2016.

56.

Clarke R. Information technology and dataveillance. Communications of the ACM 1988;31:498–512 . Also: “…the prospects of ‘surveillance capitalism’ being exploited for ulterior motives are only just starting to be understood, often to the surprise and concern of the platforms themselves. Such is the commitment to this guiding thesis that it has spawned a new doctrine: ‘dataism’, in which information flow is the ‘supreme value’.” Hankey S , Morrison J , Naik R. Data and Democracy in the Digital Age. London, UK: The Constitution Society; 2018 , at 8.

57. See note 52, Zuboff 2015, at 85.

58. See note 16, Mirowski 2018, at 178.

59. Ellis-Petersen H. India’s Covid-19 app fuels worries over authoritarianism and surveillance. *The Guardian* 2020 May 4; available at https://www.theguardian.com (last accessed 8 May 2020).

60. See a series of Covid-19 Investigations in Lewis P, Conn D, Pegg D. UK government using confidential patient data in coronavirus response. *The Guardian* 2020 Apr 12; Lewis P, Pegg D. Google executive took part in Sage meeting, tech firm confirms. *The Guardian* 2020 Apr 30; Evan R, Pegg D. Vote leave AI firm wins seven government contracts in 18 months. *The Guardian* 2020 May 4; available at https://www.theguardian.com (all last accessed 8 May 2020).

61. Morrison, S. The world’s scariest facial recognition company is now linked to everybody from ICE to Macy’s. *Vox* 2020 Feb 28; available at https://www.vox.com (last accessed 8 May 2020).

62.

Capps B. The public interest, public goods, and third party access to UK Biobank. Public Health Ethics 2012;5 :240–51.

63.

Haggerty D , Ericson E. The surveillant assemblage. British Journal of Sociology 2000;51 10.1080/0007131002001528011140886:605–22.

64.

Cinnamon J. Social injustice in surveillance capitalism. Surveillance & Society 2017;15 :609–25.

65. 345-363 Brownsword R. Knowing me, knowing you—profiling, privacy and the public interest In Hildebradt H , Gutwirth S , eds. Profiling the European Citizen: Cross-Disciplinary Perspectives. Berlin, Germany: Springer; 2008, at

66.

Koulikov M. Emerging problems in knowledge sharing and the three new ethics of knowledge transfer. Knowledge Management & E-Learning: An International Journal 2011;3:237–250 .

67. See note 6, Contreras, Knoppers 2018.

68.

Middleton A , Milne R , Thorogood A , Kleiderman E , Niemiec E , Prainsack B , et al Attitudes of publics who are unwilling to donate DNA data for research. European Journal of Medical Genetics 2018;62 10.1016/j.ejmg.2018.11.014PMC658263530476628:316–23.

69.

Zastrow M. Open science takes on the Coronavirus pandemic. Nature 2020;58110.1038/d41586-020-01246-332332909:109–10.

70. See note 6, Contreras, Knoppers 2018. The authors describe the “genomic commons” (with similar characteristics shared with “scientific disciplines of all descriptions”) as “the worldwide collection of genomic data that is generally available for public use.”

71.

Agrawal A. Sustainable governance of common-pool resources: Context, methods, and politics. Annual Reviews of Anthropology 2003;32 :243–62.

72. See note 6, Contreras, Knoppers 2018.

73.
**See note 6, Contreras, Knoppers 2018.

74. Global Alliance for Genomics and Health. A federated ecosystem for sharing genomic, clinical data. *Science* 2016;352:1278–80; also: on “controlled access” and “completely open access”, see note 41, Knoppers, Joly 2018.

75.

Saunders F. The promise of common pool resource theory and the reality of commons projects. International Journal of the Commons 2014;8 :636–56.

76.

Brown W. Neo-liberalism and the end of liberal democracy. Theory & Event 2003;7(1); doi:10.1353/tae.2003.0020.

77.

Zimmer M. “But the data is already public”: On the ethics of research in Facebook. Ethics in Information Technology 2010;12:313–25; Lewis K , Kaufman J , Gonzalez M , Wimmer A , Christakis, N. Tastes, ties, and time: A new social network dataset using Facebook.com. Social Networks 2008;30 :330–42; Kramer A , Guillory JE , Hancock, JT . Experimental evidence of massive-scale emotional contagion through social networks. PNAS 2014;111 10.1073/pnas.1320040111PMC406647324889601:8788–90.

78.

Raymond N. Reboot ethical review for the age of big data. Nature 2019;568:277 3098846610.1038/d41586-019-01164-z. Ostrom also held that: “The phenomenon known as the tragedy of the commons will occur in highly valued open-access commons where those involved and/or external authorities do not establish an effective governance regime”; Ostrom E. *Polycentric Systems as One Approach for Solving Collective-Action Problems.* Working Paper W08-6. Bloomington: Indiana University Workshop in Political Theory and Policy Analysis; 2008, at 7.

79. See note 40, Capps 2016.

80. European Commission. *Commission Staff Working Document; Accompanying the Document: Commission Recommendation on Access to and Preservation of Scientific Information* {C(2018) 2375 final} Brussels, 25.4.2018; SWD(2018) 123 final; 2018.

81. See note 40, Capps 2016.

82. See note 17, Brownsword 2009.

83. Gerrshman J. First amendment experts: Downloading hacked Clinton campaign emails not a crime. *Wall Street Journal* 2016 Oct 17; available at https://blogs.wsj.com (last accessed 8 May 2020).

84. Leetaru K. The problem isn’t Cambridge Analytica: It’s Facebook. *Forbes* 2018 Mar 19; available at https://www.forbes.com (last accessed 8 May 2020).

85. See note 6, Contreras, Knoppers 2018.

86.

Gewirth A. The community of rights In: Morscher E , Neumaier O , Simons P , eds. Applied Ethics in a Troubled World. Philosophical Studies Series, *Vol.* 73 Dordrecht, The Netherlands: Springer; 1998, at 225-235 .

87.

Chapman A. Towards an understanding of the right to enjoy the benefits of scientific progress and its application. Journal of Human Rights 2009;8:1–36 .

88.

Shaver L. The right to science and culture. Wisconsin Law Review 2010;1 :121–84.

89. “Dignity” has become a conservative protest against controversial experiments (e.g., embryo research or human cloning), leading many to decry its too prohibitive reach; Porsdam Mann S, Donders Y, Mitchell C , Bradley VJ , Chou MF , Mann M , et al Opinion: Advocating for science progress as a human right. PNAS 2018;115 10.1073/pnas.1816320115PMC620548230352870:10820–3. Although we should be rightly wary of the Dignitarian’s false claims, careful analysis of the function of dignity may also provide legitimating criteria for such experiments, too; Brownsword R. Bioethics today, bioethics tomorrow: Stem cell research and the ‘Dignitarian Alliance’. Notre Dame Journal of Law, Ethics and Public Policy 2003;17:15–52 14986644.

90.

Kramer M. Rights without trimmings In: Kramer M , Simmonds N , Steiner H , eds. A Debate Over Rights. Oxford, UK: Clarendon Press; 1998, at 7–111 .

91.

Capps B. Privacy, rights and biomedical data collections In: Kaan T , Ho C , eds. Genetic Privacy in Singapore: Science, Ethics and Law. Singapore: World Scientific: 2013, at 169–202 .

92. See note 17, Gewirth 1998.

93.

Khan A , Capps BJ , Sum MY , Kuswanto CN , Sim K . Informed consent for human genetic and genomic studies: A systematic review. Clinical Genetics 2014;86:199–206 2464640810.1111/cge.12384.

94. See note 17, Gewirth 1998.

95.

Capps B. Defining variables of access to UK Biobank: The public interest and the public good. Law, Innovation and Technology 2013;5 :113–39.

96. This is underscored by the “privacy tradeoff fallacy” that runs counter to the claim that people *are always* happy to give up their personal data in exchange for perceived benefits; Turow J, Hennessy M, Draper N. *The Tradeoff Fallacy: How Marketers are Misrepresenting American Consumers and Opening Them up to Exploitation.* Philadelphia: Annenberg School for Communications, University of Pennsylvania; 2015.

97. See note 95, Capps 2013.

98. See note 17, Gewirth, 1998, at xii.

99. See note 17, Gewirth, 1998, at xii.

100.

Tene O , Polonetsky J. Big data for all: Privacy and user control in the age of analytics. Northwestern Journal of Technology and Intellectual Property 2013;11 :239–73.

101. Wong J. Zuckerberg Says Facebook is pivoting to privacy after a year of controversies. *The Guardian* 2019 Mar 6; available at https://www.theguardian.com (last accessed 8 March 2020).

102. See note 95, Capps 2013.

103.

Capps B , Van de Eijk Y , Krahn T . Conflicts of interest in e-cigarette research: A public good and public interest perspective. Bioethics 2020;34 10.1111/bioe.1261931448428:114–22.

104.

Maurer S. Open source drug discovery: Finding a niche (or maybe several). UMKC Law Review 2007;76 :405–35; Wright B , Boettinger S. Open source in biotechnology: Open questions. Innovations, Technology, Governance, Globalisation 2006;1:43–55 .

105. OpenSAFELY conducted a unique, very rapid analysis of very large numbers of confidential medical records to understand demographics during the COVID-19 pandemic. That project’s efficacy relied on a new code that ensured a secure connection and tracked the researchers’ actions. Arguably, that permitted them to legitimately interrogate the public good, that is they accessed, without consent, the National Health Service health records of British patients as credible “public health servants” and subject to British law. The code that enabled them to do so has been made available as open source. OpenSAFELY Collaborative. OpenSAFELY: Factors associated with COVID-19-related hospital death in the linked electronic health records of 17 million adult NHS patients. *medRXiv* 2020 May 7; available at https://www.medrxiv.org/content/10.1101/2020.05.06.20092999v1.full.pdf+html (last accessed 8 May 2020).

106. Stein, S. How to restore data privacy after the coronavirus pandemic. *World Economic Forum* 2020 Mar 31; available at https://www.weforum.org/agenda/2020/03/restore-data-privacy-after-coronavirus-pandemic/ (last accessed 18 May 2020).

107. See note 95, Capps 2013.

108.

Ostrom E. Institutional arrangements for resolving the commons dilemma: Some contending approaches In: McCay B , Acheson J , eds. The Question of the Commons: The Culture and Ecology of Communal Resources. Tucson: University of Arizona Press; 1987 , at 250–65, at 250.

109. See note 12, Zuboff 2016.

